# Flower-Like Dual-Defective Z-Scheme Heterojunction g-C_3_N_4_/ZnIn_2_S_4_ High-Efficiency Photocatalytic Hydrogen Evolution and Degradation of Mixed Pollutants

**DOI:** 10.3390/nano11102483

**Published:** 2021-09-24

**Authors:** Linlin Hou, Zhiliang Wu, Chun Jin, Wei Li, Qiuming Wei, Yasi Chen, Teng Wang

**Affiliations:** 1 Guangdong Provincial Key Laboratory of Quantum Engineering and Quantum Materials, Guangdong Engineering Technology Research Center of Efficient Green Energy and Environmental Protection Materials, School of Physics and Telecommunication Engineering, South China Normal University, Guangzhou 510006, China; 2019021952@m.scnu.edu.cn (L.H.); 2019021957@m.scnu.edu.cn (Z.W.); chunjin@m.scnu.edu.cn (C.J.); 2019021930@m.scnu.edu.cn (Q.W.); yasi@m.scnu.edu.cn (Y.C.); 2 Guangdong Provincial Key Laboratory of Nuclear Science, South China Normal University, Guangzhou 510006, China; 3 School of Computer Science, South China Normal University, Guangzhou 510006, China; towangteng@263.net

**Keywords:** Z-scheme heterojunction, oxygen-doping, defect engineering, hydrogen evolution, mixed pollutants

## Abstract

Graphitic carbon nitride (g-C_3_N_4_) with a porous nano-structure, nitrogen vacancies, and oxygen-doping was prepared by the calcination method. Then, it was combined with ZnIn_2_S_4_ nanosheets containing zinc vacancies to construct a three-dimensional (3D) flower-like Z-scheme heterojunction (pCN-N/ZIS-Z), which was used for photocatalytic hydrogen evolution and the degradation of mixed pollutants. The constructed Z-scheme heterojunction improved the efficiency of photogenerated charges separation and migration, and the large surface area and porous characteristics provided more active sites. Doping and defect engineering can change the bandgap structure to improve the utilization of visible light, and can also capture photogenerated electrons to inhibit recombination, so as to promote the use of photogenerated electron-hole pairs in the photocatalytic redox process. Heterojunction and defect engineering synergized to form a continuous and efficient conductive operation framework, which achieves the hydrogen production of pCN-N/ZIS-Z (9189.8 µmol·h^−1^·g^−1^) at 58.9 times that of g-C_3_N_4_ (155.9 µmol·h^−1^·g^−1^), and the degradation rates of methyl orange and metronidazole in the mixed solution were 98.7% and 92.5%, respectively. Our research provides potential ideas for constructing a green and environmentally friendly Z-scheme heterojunction catalyst based on defect engineering to address the energy crisis and environmental restoration.

## 1. Introduction

Energy and the environment have been important issues in the history of human development. At present, many studies have reported that hydrogen is precipitated by electrolysis of water, but this method is energy-intensive and costly and is not conducive to mass production. Therefore, exploring low-cost, simple and environmentally friendly methods to produce hydrogen has become an important research topic [1,2]. At the same time, with the progress of human society and the development of the industrial age, the use of antibiotics has increased due to the emergence of a variety of new diseases, and the entry of antibiotics into water will pose risks to human health and ecosystems [3,4,5,6,7]. Among them, metronidazole (MNZ) (Figure S1a) is one of the most widely used antibiotics. Different contents were found in water, ranging from 1 to 10 ng/L [8,9]. Traditional wastewater treatment methods cannot completely remove MNZ [10,11,12,13,14]. A large number of research reports can prove that semiconductor-based photocatalysis has been regarded as a promising strategy to remove MNZ [9]. In addition, due to the random discharge of industrial wastewater, dye wastewater has become one of the common pollution sources in the water environment. Dye wastewater pollution seriously affects the balance of the ecosystem and natural environment [15,16,17]. The most typical dye in wastewater is methyl orange (MO) (Figure S1b) [16,18,19,20,21]. Therefore, it is urgent to find green and clean technologies to solve the energy crisis and water pollution problems.

For decades, many researchers have studied photocatalytic technology based on semiconductor materials to address environmental pollution and energy regeneration. Carey et al. studied the photocatalytic degradation of polychlorinated biphenyls by titanium dioxide materials in 1976 [22]. Guo et al. studied the phosphorus-doped carbon nitride for photocatalytic hydrogen evolution under visible light, and the hydrogen production rate reached 670 µmol·h^−1^·g^−1^ [23]. Ahmad et al. studied the photocatalytic hydrogen evolution mechanism of ZnCdS nanocrystals under alkaline conditions, and the hydrogen production rate reached 21.1 mmol·h^−1^·g^−1^ [24]. Semiconductor-based photocatalytic technology has been regarded as a promising strategy for energy regeneration and degradation of harmful organic pollutants. Unfortunately, most of the photocatalysts studied are wide bandgap semiconductors that cannot effectively absorb visible light. In addition, most of the currently reported catalysts cannot degrade mixed pollutants. Hence, it is a challenging problem to explore new and powerful visible light-driven catalyst to efficiently produce hydrogen and degrade mixed pollutants. However, there are few reports on multifunctional catalyst materials. The strategies to improve catalyst performance include precious metal deposition, ion doping and construction of heterojunctions [25,26]. Since Bard et al. first published an article on Z-scheme heterojunction for water splitting [27], this has attracted the interest of researchers [28,29,30]. According to the reported structure of a heterojunction, it can be found that the Z-scheme can not only increase light absorption of the two effective semiconductors, but also improve the redox capacity and photogenerated charge separation efficiency [30,31,32]. However, the selection of composition with matching semiconductors is an important issue in a Z-scheme. Therefore, it is very important to choose a suitable photocatalyst system and use photocatalytic technology to solve energy and environmental pollution problems.

At present, two-dimensional (2D) graphitic carbon nitride (g-C_3_N_4_) is a typical non-metallic and non-toxicity polymeric photocatalyst (CN), which has attracted tremendous attention because of its characteristics, such as excellent stability, favorable band positions, etc. [33]. Nevertheless, it also has lots of deficiencies such as low surface area, narrow light absorption range, etc. [34,35]. Therefore, it is necessary to improve the photocatalytic performance by modifying it. Through research, oxygen doping can promote the separation of charges on the triazine ring, causing light to excite electrons, thereby enhancing the photocatalytic activity of g-C_3_N_4_ [36]. Jiang et al. reported that oxygen doping and nitrogen defects in g-C_3_N_4_ can change the electronic transition, thereby inhibiting charge recombination. Finally, the hydrogen evolution rate of the modified g-C_3_N_4_ can be increased by six times [37]. The introduction of nitrogen defects can adjust the energy band position of the catalyst and improve the efficiency of carrier separation and transfer [38]. Kong et.al reported that the N vacancy increased the reaction rate of g-C_3_N_4_ photocatalyzed 2-propanol to acetone by 9.6 times [39]. Therefore, composite photocatalyst technology is one of the ways to improve the photocatalytic performance of g-C_3_N_4_. Zinc indium sulfide (ZnIn_2_S_4_) has attracted great attention among researchers because of its peculiar electronic structure excellent and visible light absorption range, etc. [40,41,42]. Qin et al. studied the photocatalytic hydrogen evolution performance of ZnIn_2_S_4_/gC_3_N_4_ (6095.1 μmol·h^−1^·g^−1^) and the electron transport mechanism in the photocatalytic system [43]. Gao et al. used covalent bonds to couple ZnIn_2_S_4_ and g-C_3_N_4_ nanosheets for photocatalytic hydrogen production, and the hydrogen production rate of ZnIn_2_S_4_/gC_3_N_4_ was about 15.1 times that of pure g-C_3_N_4_ [44]. Therefore, in this paper, g-C_3_N_4_ with nitrogen vacancies (pCN-N) and ZnIn_2_S_4_ with zinc vacancies (ZIS-Z) are selected as composite catalyst materials to achieve photocatalytic hydrogen evolution and degradation of mixed pollutants. Zinc vacancies can increase the charge density, improve the separation and transport efficiency of carriers, and can also increase the light absorption capacity of ZnIn_2_S_4_ [41]. The composite catalyst system is beneficial to improve the separation and transfer of photo-generated electrons and holes so as to improve the photocatalytic performance.

**Figure 1:**
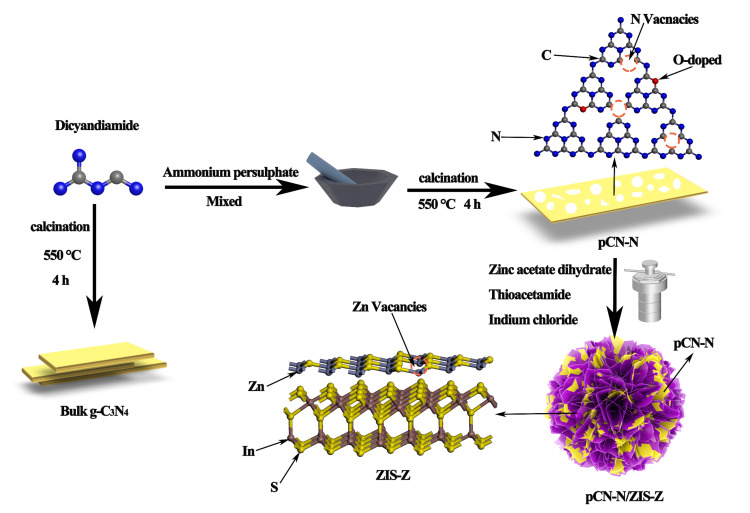
The synthesis process of CN, pCN-N, ZIS-Z, pCN-N/ZIS-Z.

Based on the above analysis, we report a novel oxygen-doped g-C_3_N_4_ and ZnIn_2_S_4_ Z-scheme heterojunction photocatalyst based on defect engineering (pCN-N/ZIS-Z), which achieves dual-functional high-efficiency catalysis. The corresponding experimental synthesis process is shown in Figure 1. The pCN-N is obtained by grinding dicyandiamide and ammonium persulfate, and then heating it to 550 °C for 240 min in a muffle furnace. The ZnIn_2_S_4_ precursor solution containing a certain amount of pCN-N was placed in a stainless-steel autoclave and kept at 200 °C for 18 h. When the reaction system was cooled to room temperature, the pCN-N/ZIS-Z composite material with double defects was successfully synthesized. Also, the relationship between the structure of the catalyst material and the catalytic performance was systematically analyzed through different test and characterization results. Finally, the cyclic stability and possible photocatalytic mechanism of this research in the photocatalytic system are proposed.

## 2. Materials and Methods

### 2.1. Materials

Dicyandiamide (C_2_H_4_N_4_), Metronidazole (C_6_H_9_N_3_O_3_) and Indium chloride (InCl_3_) were purchased from Shanghai Macklin Biochemical Co., Ltd. Ethanol (Shanghai, China) absolute (CH_3_CH_2_OH), Zinc acetate dihydrate (Zn(CH_3_COO)_2_·2H_2_O), Methyl orange (C_14_H_15_N_3_NaO_3_S), and Thioacetamide (TAA) were purchased from Tianjin Damao Chemical Reagent Factory (Tianjin, China). Hydrochloric acid (HCl, 36.0~38.0%) (Standard operation, pay attention to safety) and Ammonium persulphate ((NH_4_)_2_S_2_O_8_) were purchased from Guangdong Guangshi Reagent Technology Co., Ltd. (Guangzhou, China). All reagents used in the experiment did not need to be purified.

### 2.2. Synthesis of Porous 2D Graphitic Carbon Nitride Nanosheets with Nitrogen Vacancies and Oxygen Doping

Dicyandiamide (10 g) and ammonium persulphate (5 g) were ground and then heated to 550 °C for 240 min. The obtained precursor (0.1 g) was sonicated in deionized water (200 mL) for 30 min. Under magnetic stirring, hydrochloric acid (3 mL) was added to the mixed solution for exfoliation for 4 h. Finally, centrifuge washing was used to obtain the desired material (named pCN-N). For comparison, the dicyandiamide was heated to 550 °C and maintained for 240 min in the muffle furnace to obtain bulk g-C_3_N_4_ (named CN).

### 2.3. Synthesis of 2D ZnIn_2_S_4_ Nanosheets with Zn Vacancies

Zinc acetate dihydrate (0.25 mmol), Indium chloride (0.5 mmol), Thioacetamide (2 mmol) and 70 mL deionized water were mixed and stirred on a constant magnetic stirrer for 30 min. We poured the mixture into a stainless-steel autoclave and kept it at 200 °C for 18 h. Finally, centrifuge washing was used to obtain the desired material (named ZIS-Z). For comparison, the same experimental method was used to prepare ZnIn_2_S_4_ nanosheets without zinc vacancies (named ZIS) with the only difference being that the temperature is 180 °C.

### 2.4. Synthesis of pCN-N/ZIS-Z Nanocomposite Photocatalyst

Zinc acetate dihydrate (0.25 mmol), indium chloride (0.5 mmol), thioacetamide (2 mmol) and 70 mL deionized water were mixed and stirred on a constant magnetic stirrer for 30 min, afterwards the pCN-N (0.1 g) was added into above mixed solution. We poured the mixture into a stainless-steel autoclave and keep it at 200 °C for 18 h. Finally, the nanocomposite material g-C_3_N_4_/ZnIn_2_S_4_ was obtained by centrifugal washing (named pCN-N/ZIS-Z).

### 2.5. Characterization Methods

We used an X-ray diffractometer (XRD, BRUKER, Karlsruhe, Germany) to measure the crystal structure of the catalyst. We used a Nicolet 6700 to obtain Fourier transform infrared spectroscopy (FTIR, American Thermoelectric Nicolet, Madison, WI, USA) information. We obtained scanning electron microscope (SEM, FEI, Hillsboro, OR, USA) images through a ZEISS Gemini 500. We obtained transmission electron microscopy (TEM, FEI, Hillsboro, OR, USA) images through a FEI Talos F200X. The pore size and specific surface area of the catalyst were measured by a gas adsorption device (ASAP2020, Micromeritics, Shanghai, China) at 77 K based on the N_2_ adsorption-desorption isotherm measurement. The chemical bonds of the catalyst were studied through an X-ray photoelectron spectrometer (Thermo Fisher, XPS, Shanghai, China). We obtained photoluminescence (PL) spectra through a Hitachi F-4500 fluorescence spectrop hotometer (Tokyo, Japan). We used a Shimadzu 2550 PC spectrophotometer (Kyoto, Japan) to obtain ultraviolet−visible (UV−vis) diffuse reflection spectra. Using CHI660B electrochemical workstation (Shanghai, China) for photoelectrochemical measurements. We used a Brooke A300 to test electron paramagnetic resonance (EPR, BRUKER, Karlsruhe, Germany) of the catalyst. HPLC data were obtained with a Kejie LC-600 (Palo Alto, CA, USA).

### 2.6. Photocatalytic Activity

To evaluate the photocatalytic activities of samples, MO (20 mg/L), MNZ (30 mg/L) and the mix solution of MO (3 mg/L) and MNZ (30 mg/L) (named MO/MNZ) were degraded under visible light (λ ≥ 420 nm) at room temperature. First, the catalytic system reached the adsorption equilibrium under dark conditions. Then we turned on the xenon lamp light source to perform photocatalytic degradation experiments under visible light. During the period of illumination, we took out 1.5 mL of the reaction solution at the same time intervals and centrifuged it. We used an ultraviolet-visible spectrophotometer and HPLC to measure the remaining substances.

In order to expand the application range of the catalyst, this paper also studied the hydrogen production performance of pCN-N/ZIS-Z. The light source was a 300 W xenon lamp (Equipped with cut-off filter, λ ≥ 420 nm). The photocatalytic hydrogen production experiment was carried out at room temperature for 5 h. Using an Agilent 7890B model gas chromatograph, the cumulative gas output was collected every 60 min. The catalyst (50 mg) was dispersed in 100 mL of triethanolamine-containing (TEOA) aqueous solution as a sacrificial reagent and 1wt% Pt was loaded on the catalyst in situ as a promoter. It was degassed for 20 min before irradiation to remove dissolved oxygen. Throughout the reaction process, continuous magnetic stirring was performed to maintain the uniform dispersion of the reaction mixture. The apparent quantum yield (AQY) was measured by inserting an appropriate band-pass filter (365 nm, 420 nm, 450 nm, 500 nm) on the light source. Other conditions were similar to those for hydrogen measurement.

## 3. Results

### 3.1. Photocatalyst Characterization

**Figure 2:**
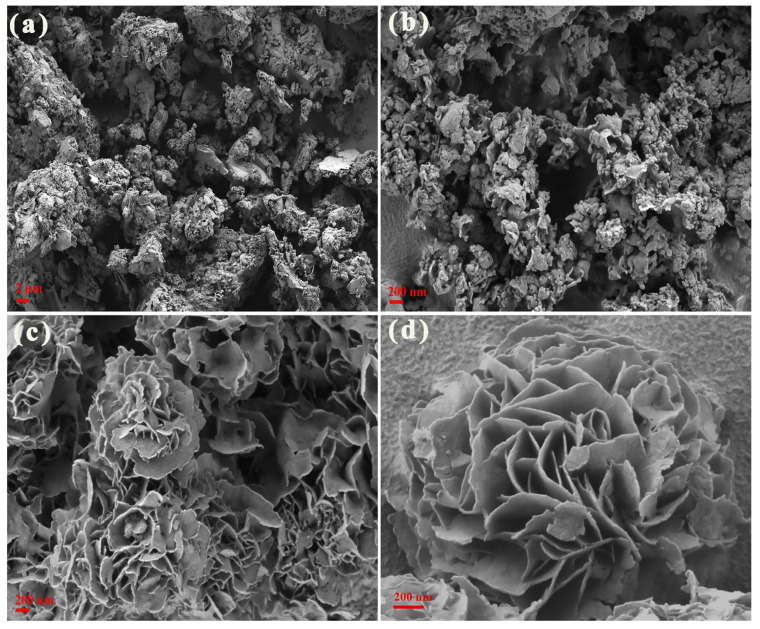
SEM images of bulk CN (**a**), pCN-N nanosheets (**b**), ZIS-Z nanosheets (**c**) and pCN-N/ZIS-Z (**d**).

**Figure 3:**
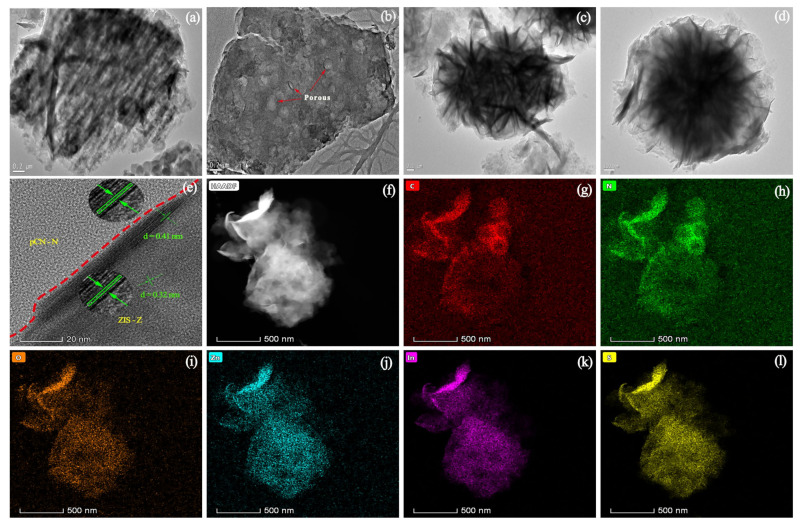
TEM images of bulk CN (**a**), pCN-N nanosheets (**b**), ZIS-Z nanosheets (**c**) pCN-N/ZIS-Z nanocomposite (**d**), HRTEM image of pCN-N/ZIS-Z nanocomposite (**e**), and elemental mapping images of pCN-N/ZIS-Z (**f**–**l**).

SEM and TEM were used to obtain the microscopic morphology and structure of bulk CN, pCN-N nanosheets, ZIS-Z nanosheets, and pCN-N/ZIS-Z nanocomposite. Figure 2a shows bulk CN presents an irregular bulk structure. Compared with CN, pCN-N presents a thin flaky shape, and the surface is relatively smooth (Figure 2b). The modified CN has a larger specific surface area. Figure 2c shows the ZIS-Z nanosheets stacked into a flower-like morphology. As depicted in Figure 2d, pCN-N and ZIS-Z nanosheets are tightly combined to form a 3D flower-like nanocomposite. The morphology engineering of stacked nanosheets expands the specific surface area of the composite. Figure 3a illustrates that CN is a bulk structure, but the modified pCN-N shows a thin and porous morphology (Figure 3b), which is consistent with the SEM morphology observation. It can be clearly observed that the ZIS-Z nanosheets are stacked on each other to constitute a flower-like morphology (Figure 3c). Figure 3d shows the ZIS-Z is covered by very thin nanosheets, which is attributed to the pCN-N embedded in the flower-like ZIS-Z. The close contact between pCN-N and ZIS-Z expands the specific surface area of the composite. It is well known that a large specific surface area is beneficial to provide more active sites for photocatalysis and efficient transport paths for reactants and products. The close contact interface between pCN-N and ZIS-Z can also be seen in Figure 3e. Additionally, the lattice spacing 0.32 nm and 0.41 nm represent the (102) and the (006) crystal plane of ZnIn_2_S_4,_ respectively [45]. Figure 3f–l illustrates the C, N, O, Zn, In, S in the pCN-N/ZIS-Z. From Figure 3i, it can be proved that oxygen-doping has occurred. According to the above data, pCN-N and ZIS-Z can be considered to have been successfully compounded and may form a heterojunction.

**Figure 4:**
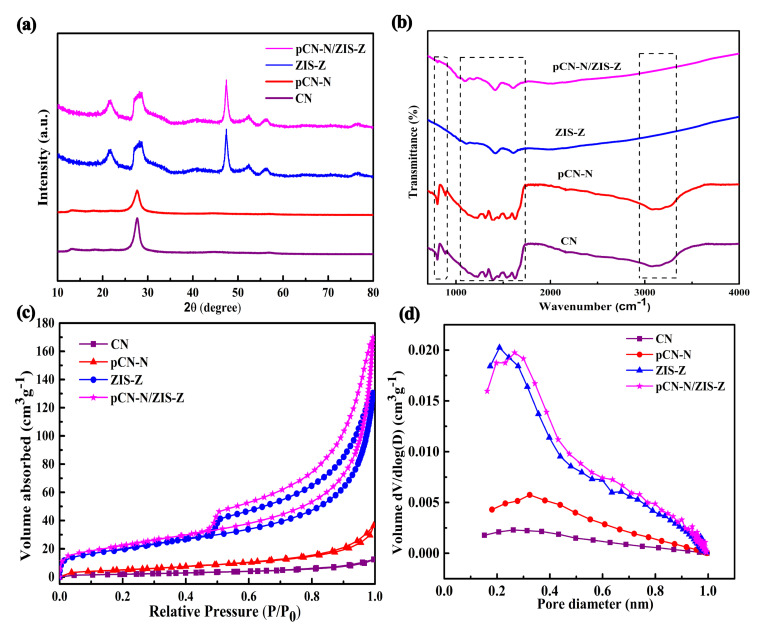
XRD patterns (**a**), FTIR spectra (**b**), surface area (**c**), and pore size distribution (**d**) of CN, pCN-N, ZIS-Z, and pCN-N/ZIS-Z catalyst.

Figure 4a shows the XRD diagrams of different catalysts. The two striking characteristic diffraction peaks of CN and pCN-N around 12.8° and 27.6° correspond to the (100) and (002) planes, which denote the in-planar structurally repeated motifs of triazine rings and the interlayer reflection of the graphite structure, respectively [38,46]. The intensity of pCN-N becomes weaker than bulk CN at 27.6°, which is possibly due to the introduction of nitrogen vacancies and Figure S3a illustrates that pCN-N contains nitrogen vacancies. As for ZIS-Z and pCN-N/ZIS-Z, the six striking characteristic diffraction peaks can be observed at 21.6°, 27.7°, 40.3°, 47.4°, 52.3° and 56.2°, corresponding to the (006), (102), (108), (110), (116) and (202) match with ZnIn_2_S_4_ (JCPDS No. 72-0773) [43]. There is almost no characteristic peak of pCN-N in pCN-N/ZIS-Z, because the diffraction peak of pCN-N (002) plane overlaps with the diffraction peak of the ZIS-Z (102) plane, and the crystallinity of ZIS-Z is higher than that of pCN-N. The FTIR spectra were adopted to analyze the chemical structure characteristics of different catalysts. As illustrated in Figure 4b, CN has three distinct sections of characteristic absorption bands. The broad peak in the range of 3000–3500 cm^−1^ are assigned to the N–H stretching vibrations of –NH_x_ groups (–NH_2_ or NH) and O–H stretching vibrations of surplus hydroxyl groups or absorbed water molecules [47]. There are some strong bands between 1200 cm^−1^ and 1700 cm^−1^ associated with the stretching vibration of C=N and C–N heterocycles. Moreover, the obvious bands at 810 cm^−1^ were derived from the characteristic breathing-vibration of the tris-triazine scheme [48]. For ZIS-Z, it can be observed that there are characteristic peaks at 1615 cm^−1^ and 1396 cm^−^^1^, which are due to water molecules and hydroxyl groups physically adsorbed on the surface [41]. Compared with ZIS-Z, the composite has the characteristic peak around 810 cm^−^^1^, which is due to the superposition of the characteristic peak of pCN-N (810 cm^−1^) and spectra of ZIS-Z. Figure 4c,d shows that all catalyst show a type-Ⅳ isotherm with a H3 hysteresis loop, indicating the existence of a mesoporous structure [49]. The enlarged N_2_ adsorption-desorption isotherms of CN and pCN-N are shown in Figure S2. Compared with CN, the hysteresis loops of pCN-N, ZIS-Z and pCN-N/ZIS-Z move to areas with lower relative pressure, and the area of the hysteresis loop increases, indicating that the mesopores are larger than CN. Table S1 shows the surface area of the modified pCN-N increased from 7.837 m^2^/g to 19.843 m^2^/g. Morphological changes can also be confirmed by TEM images. Additionally, the change in pore size from 9.783 nm to 11.360 nm also confirmed the formation to be porous. The specific surface area of pCN-N/ZIS-Z (81.848 m^2^/g) is larger than that of ZIS-Z (74.691 m^2^/g) and pCN-N (19.843 m^2^/g). The modified composite nanomaterials have a larger surface area, and it is beneficial to provide more active sites for the photocatalytic process while providing an efficient transmission path between reactants and products.

**Figure 5:**
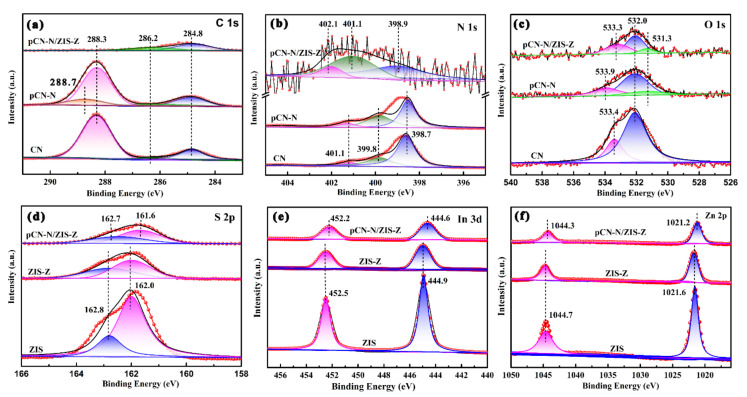
XPS spectroscopy spectra of CN, pCN-N, ZIS-Z, pCN-N/ZIS-Z nanocomposite. C 1s (**a**), N 1s (**b**), O 1s (**c**), S 2p (**d**), In 3d (**e**), and Zn 2p (**f**).

We used XPS to study the structure and surface element composition of different catalysts. The C 1s spectra of CN, pCN-N and pCN-N/ZIS-Z in Figure 5a show three main characteristic peaks at 284.8 eV, 286.2 eV and 288.3 eV, which can be attributed to adventitious carbon species(C–C), sp^3^-bonded carbon C–NH_x_ (*x* = 1, 2) on the edges of heptazine units and sp^2^-bonded carbon in (N–C=N), respectively [50]. It can be found that pCN-N has a new characteristic peak at 288.7 eV compared to CN, which corresponds to C=O [47], which can be attributed to oxygen-doping. At 288.3 eV, the characteristic peak of pCN-N is weaker than that of CN, which is due to the nitrogen defect in pCN-N. In addition, Figure S3a also proves the existence of nitrogen vacancies in pCN-N. It can be found that the C 1s spectra for pCN-N/ZIS-Z involve only two dominating characteristic peaks. On the one hand, it may be that the crystal phase of pCN-N is weaker than that of ZIS-Z, which is consistent with the results of XRD and FTIR. On the other hand, it may be that the hydrothermal process has further formed nitrogen vacancies and reduced peak intensity. Figure 5b shows the N 1s spectra of CN and pCN-N have three types of nitrogen peak at binding energies of 398.7 eV, 399.8 eV and 401.1 eV, which are ascribed to sp^2^-hybridized nitrogen in triazine ring(C=N–C), the tertiary nitrogen (C)_3_-N in the aromatic rings and N–H groups_,_ respectively [51]. However, the three characteristic peaks of pCN-N/ZIS-Z appear at 398.9 eV, 401.1 eV and 402.1 eV, which are shifted in the direction of higher binding energy relative to CN and pCN-N, indicating a decrease in electron density. In fact, the N species of the pCN-N component in the pCN-N/ZIS-Z composite is an electron donor, and the electrons of pCN-N are turned to ZIS-Z. In addition, through the shift of the N 1s peak in the composite material, it can be considered that there is a strong interfacial interaction between pCN-N and ZIS-Z [52]. Figure 5c shows the main characteristic peaks of the O 1s spectrum. The peak of pure CN at a binding energy of 533.4 eV is attributed to the formation of surface hydroxyl groups (O-H) due to the absorption of water on the surface [53]. Second, the peak at the binding energy of 532.0 eV is due to the O–N bond formed during the thermal polymerization of dicyandiamide [54]. However, it can be found that compared with pure CN, both pCN-N and pCN-N/ZIS-Z have a new O 1s spectrum, which is located at the binding energy of 531.3 eV. This phenomenon can be attributed to the O–C–N and O–C species formed by oxygen doping in the crystal lattice [55], which proves that the oxygen atom is combined with the carbon atom in the CN aromatic heterocycle. Moreover, it can be observed that the peak of pCN-N at 533.9 eV and the peak of pCN-N/ZIS-Z at 533.3 eV are slightly shifted relative to the peak of pure CN at 533.4 eV. This may be due to the oxygen-doping in pCN-N and the chemical interaction between pCN-N and ZIS-Z in the nanocomposite material pCN-N/ZIS-Z, which results in the change of binding energy. From the comparison of N 1s and O 1s spectra, it is found that the characteristic peaks of pCN-N/ZIS-Z have a certain offset relative to CN and pCN-N. This may be caused by the formation of a heterojunction due to the interaction between pCN-N and ZIS-Z in close contact. The formation of heterojunction changes the electronic structure, and ultimately causes the position of the characteristic peaks to shift. The S 2p XPS spectrum (Figure 5d) reveals the S 2p_1/2_ and S 2p_3/2_ of binding energies at 162.8 eV and 162.0 eV, respectively [56]. The two peaks of binding energy at 452.5 eV and 444.9 eV correspond to In 3d_3/2_ and In 3d_5/2_, respectively (Figure 5e) [57]. The Zn 2p XPS spectrum shows the binding energies of Zn 2p_1/2_ at 1044.7 eV and Zn 2p_3/2_ at 1021.6 eV (Figure 5f) [58]. By comparison, it was found that the characteristic peak areas of the S 2p, In 3d and Zn 2p spectra of pCN-N/ZIS-Z were significantly reduced compared to ZIS and ZIS-Z. It can be further considered that there is a chemical interaction between pCN-N and ZIS-Z in nanocomposites. Compared with the pure ZIS-Z, the S 2p, In 3d and Zn 2p spectra in the nanocomposite there is lower binding energy. This can be attributed to the close contact between pCN-N and ZIS-Z, some electrons will be transferred from pCN-N to ZIS-Z. That is, the electron density of ZIS-Z in the composite material increases, resulting in a decrease in the binding energy of pCN-N/ZIS-Z. Thus, it is further proved that there is an electronic interaction between pCN-N and ZIS-Z in the nanocomposite, which promotes the separation and transfer of photogenerated electron-hole pairs. According to the XPS analysis, it can be concluded that there is a strong interfacial coupling effect between the nitrogen-rich and oxygen-doped pCN-N and the zinc vacancy ZIS-Z (see Supplementary Materials Figure S3b for EPR analysis of zinc vacancies) to form a heterojunction structure [59,60], which further confirms the conclusions of XRD and FTIR. The formation of the heterojunction is beneficial to promote the separation and transfer of photogenerated electron and hole pairs, which is beneficial to improving the photocatalytic performance of pCN-N/ZIS-Z.

### 3.2. Photocatalytic Hydrogen Evolution

**Figure 6:**
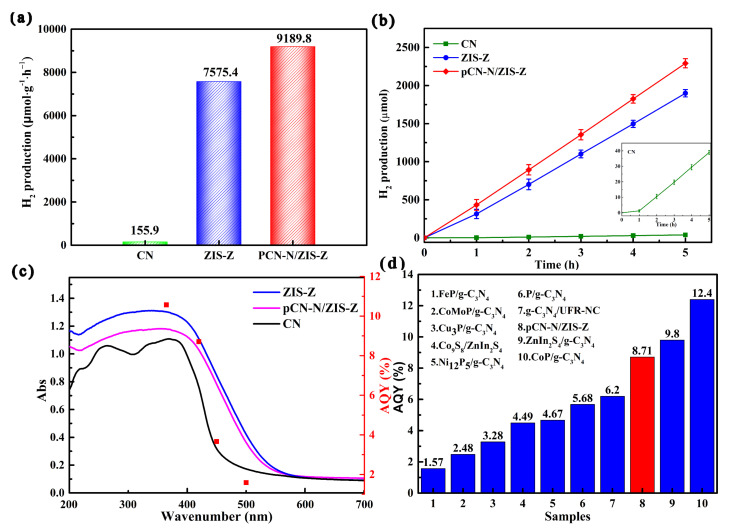
Photocatalytic hydrogen evolution activities of different catalysts (**a**), and the process of producing hydrogen by different catalysts (Insert is an enlarged view of pCN-N) (**b**), wavelength-dependent AQY of H_2_ production over pCN-N/ZIS-Z (**c**), comparison of the AQY of pCN-N/ZIS-Z with other similar research results at 420 nm (1 [61], 2 [62], 3 [63], 4 [44], 5 [64], 6 [65], 7 [66], 9 [44], 10 [67]) (**d**).

Photocatalysis experiments were performed to explore the hydrogen evolution performance of pCN-N/ZIS-Z. As depicted in Figure 6a, the hydrogen production of pCN-N/ZIS-Z (9189.8 µmol·h^−1^·g^−1^) is 58.9 times that of CN (155.9 µmol·h^−1^·g^−1^) and 1.2 times that of ZIS-Z (7575.4 µmol·h^−1^·g^−1^). Figure 6b shows the hydrogen production of different catalysts over time, and the order is pCN-N/ZIS-Z (2297.5 µmol) > ZIS-Z (1893.9 µmol) > CN (39.0 µmol) (cycle experiments in Figure S4). Cycling experiments can prove the repeatability and stability of pCN-N/ZIS-Z in the photocatalytic hydrogen production experimental system. Figure 6c shows wavelength-dependent apparent quantum yield (AQY) of photocatalytic hydrogen production. It can be observed that the wavelength-dependent AQY is similar to that of ZIS-Z and pCN-N/ZIS-Z. The wavelength-dependent AQY trend is different from that of CN. It can be inferred that the photogenerated electrons involved in the hydrogen production reaction come from ZIS-Z. The AQY reaches 10.57% at 365 nm, and 1.59% AQY can be obtained at 500 nm, which implies that pCN-N/ZIS-Z has excellent catalytic activity in the range of 420 nm to 500 nm. Figure 6d shows the comparison of the AQY of pCN-N/ZIS-Z with the results of other similar studies at 420 nm. It can be found that the AQY of PCN-N/ZIS-Z is higher than most similar types of catalyst. The comparison of similar hydrogen evolution experiments is shown in Table 1. It can be observed that the amount of hydrogen evolved from PCN-N/ZIS-Z is much higher than that of most catalysts. Through hydrogen evolution experiments, it was found that the formation of heterojunctions and the introduction of defects are beneficial to the separation and efficient transfer of photogenerated electron-hole pairs to achieve efficient hydrogen evolution. Through research, it was found that pCN-N/ZIS-Z has excellent photocatalytic hydrogen evolution performance. This provides a potential research direction to solve the energy crisis, but still needs continuous exploration.

### 3.3. Photocatalytic Degradation Performance

Firstly, this article explores the degradation performance of the catalyst on MO and MNZ, respectively. Secondly, this article explores the degradation performance of the catalyst on the mixture of MO and MNZ (MO/MNZ).

**Figure 7:**
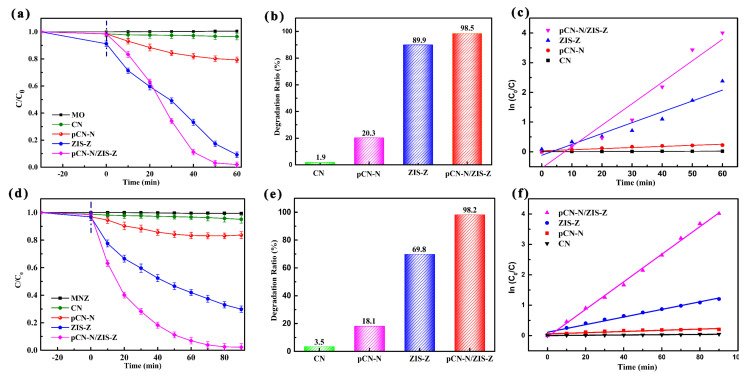
Photocatalytic activities of CN, pCN-N, ZIS-Z and pCN-N/ZIS-Z toward the degradation of MO (**a**–**c**), MNZ (**d**–**f**).

Figure 7a shows the reaction system of photocatalytic degradation of MO (20 mg/L). The pCN-N/ZIS-Z nanocomposite exhibits the highest removal rates of MO with a sequence of pCN-N/ZIS-Z > ZIS-Z > pCN-N > CN. Figure 7b reveals degradation rates of CN, pCN-N, ZIS-Z, pCN-N/ZIS-Z to MO is 1.9%, 20.3%, 89.9%, 98.5%, respectively. The electronic energy band state of CN consists of δ band (sp^3^ C–N bonding), π band (sp^2^ C–N bonding) and the lone pair (LP) state of the bridge nitride atoms. The π band state and the LP state are changed due to the introduction of defects and oxygen-doping, which further change the electron transport path and inhibit the recombination of electron-hole pairs [37,72,73]. Compared with pCN-N, the photocatalytic degradation efficiency of pCN-N/ZIS-Z is increased by 78.2%, which is attributed to the formation of heterojunctions and the increase of catalytically active sites. Figure 7c is obtained according to the equation ln(C_0_/C) = kt. Figure 7c illustrates that the pCN-N/ZIS-Z nanocomposite shows maximum reaction rate constants (0.0722 min^−1^) of 240.7, 19.5 and 1.97 times that of CN (0.0003 min^−1^), pCN-N (0.0037 min^−1^) and ZIS-Z (0.0366 min^−1^), respectively. Similarly, during the following catalytic photodegradation of MNZ (30 mg/L), the pCN-N/ZIS-Z nanocomposite also exhibits the highest removal rates with a sequence of pCN-N/ZIS-Z > ZIS-Z > pCN-N > CN under the visible light irradiation within 90 min (Figure 7d). Figure 7e shows the degradation rates of CN, PCN-N, ZIS-Z, pCN-N/ZIS-Z to MNZ is 3.5%, 18.1%, 69.8%, 98.2%, respectively. Figure 7f is obtained according to the equation ln(C_0_/C) = kt. As shown in Figure 7f, the pCN-N/ZIS-Z nanocomposite shows maximum reaction rate constants (0.0454 min^−1^) of 90.8, 22.7 and 3.6 times that of CN (0.0004 min^−1^), pCN-N (0.0020 min^−1^) and ZIS-Z (0.0125 min^−1^), respectively.

**Figure 8:**
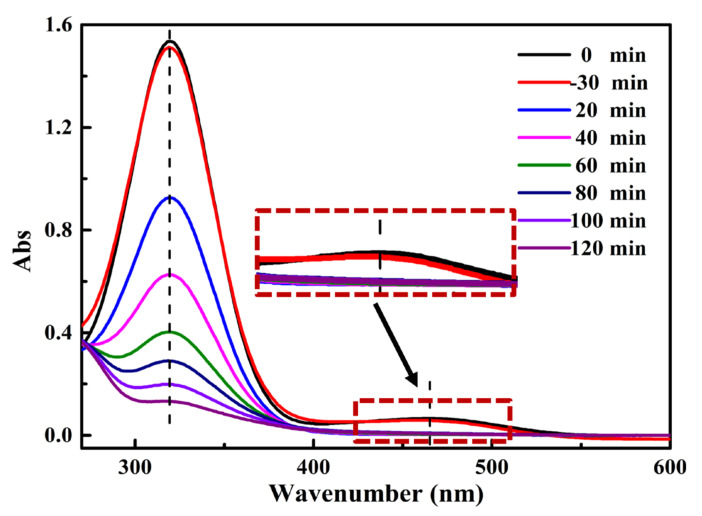
Photocatalytic degradation of mixed pollutants.

On the premise of ensuring degradation efficiency, we found that the maximum concentration of the pCN-N/ZIS-Z degradation mixture is MO (3 mg/L)/MNZ (30 mg/L). Figure 8 shows the process of pCN-N/ZIS-Z degrading MO/MNZ under visible light for 120 min. The absorption peaks of MO and MNZ are at 464 nm and 320 nm, respectively. The degradation rates of MO and MNZ are 98.7% and 92.5%, respectively. It was found that MO was completely degraded within 20 min. It can be concluded that pCN-N/ZIS-Z can not only exhibit high-efficiency hydrogen evolution performance, but also efficiently degrade mixed pollutants due to abundant defects and the formation of heterojunctions. The molecular structures of MNZ and MO are shown in Figure S1. Actually, the electron-donating conjugation effect of the amino group in the molecular structural formula of methyl orange and the hydroxyl group in the molecular structural formula of metronidazole is greater than the electron-withdrawing induction effect. However, ZIS-Z has a flower-like structure with a large surface area that can fully contact degraded pollutants in the water. In addition, the Zn vacancies in ZIS-Z can be used as electron traps to enrich electrons to reduce the surface electrostatic potential [60]. Therefore, the electron-donating groups on the surface of methyl orange and metronidazole can be combined with the electron traps on the surface of ZIS-Z to enhance the ability of the catalyst in order to adsorb and contact pollutants, and further improve the catalytic degradation performance.

### 3.4. Possible Photocatalytic Degradation Pathways of Pollutants

Figure S5 shows the HPLC spectra of MO and MNZ. Figure S5a illustrates that MO is finally degraded within 90 min. At 9 min, the characteristic peak intensity of MO decreases with the increase of the reaction time. At the same time, intermediate products are formed at 5 min, but they are completely degraded in the end. Figure S5b illustrates that MNZ is almost completely degraded within 90 min. At 5 min, the characteristic peak intensity of MNZ decreases with the increase of the reaction time, and at the same time, intermediate products are formed at 1.5~3 min and the peak intensity becomes stronger with the increase of the reaction time. The conclusion from the above HPLC data is consistent with the UV–vis data analysis result. The final degradation products of MO may be water and carbon dioxide. Figure S6 shows the Raman spectra of MNZ solution before and after degradation. As shown in Figure S6, the intensity of the peak at 1637 cm^−1^ gradually weakened during the catalytic process, and the peak at 1680~1620 cm^−1^ is due to C=C stretching, so it can be inferred that the C=C bond of metronidazole is destroyed. It can be considered that the degradation path of metronidazole is ring-opening.

### 3.5. Free Radical Recognition and Recyclability

**Figure 9:**
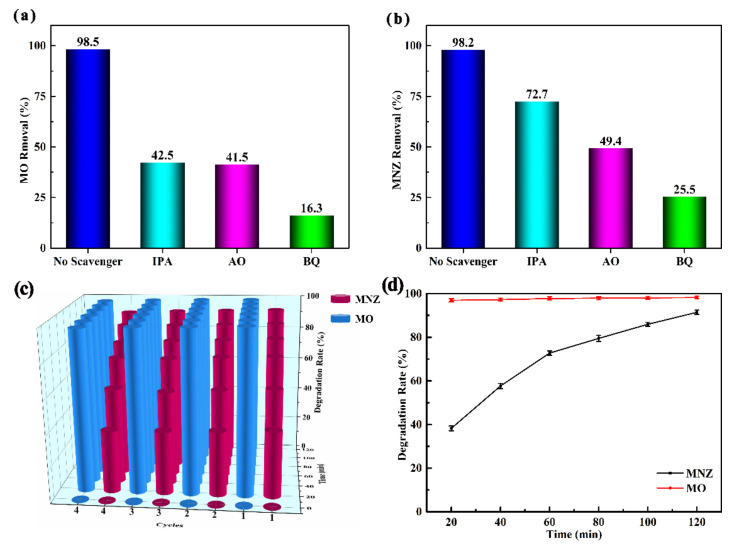
The effect of radical scavengers (IPA, AO, and BQ) on the degradation of MO (**a**) and MNZ (**b**). Photocatalytic degradation cycle (**c**) and parallel experiments (**d**) of MO/MNZ.

Figure 9a, b reveals the degradation rate of MO and MNZ after three kinds of free radical scavenger were added into the photocatalytic degradation system, respectively. Therein, IPA, AO and BQ were applied to capture OH, h^+^ and **·**O_2_^−^, respectively [74,75]. Obviously, the degradation rate of MO drops from 98.5% to 42.5%, 41.5% and 16.3%, respectively (Figure 9a). Analogously, the removal rate of MNZ drops from 98.2% to 72.7%, 49.4% and 25.5%, respectively (Figure 9b). We can conclude that **·**O_2_^−^ is most important in the photocatalytic disintegration of MO and MNZ with a sequence of **·**O_2_^−^ > h^+^ >**·**OH. Considering the practicability of the catalyst, it is necessary to carry out cycle experiments. Figure 9c shows the stability of pCN-N/ZIS-Z during the photocatalytic degradation reaction of MO/MNZ. In the four cycles, the degradation rates of MNZ at 120 min are 92.5%, 91.8%, 91.2%, and 90.3%, respectively. The degradation rates of MO at 120 min are 98.7%, 98.5%, 98.1%, and 97.4%, respectively. The reduction of the degradation rate may be due to the loss of catalyst recovery process. In order to prevent accidental errors and ensure the reliability of experimental data, four parallel experiments were carried out (Figure 9d). It can be seen from Figure 9d that the experimental data have slight fluctuations, so the observed data are reliable. Based on the above experimental data it can be concluded that the pCN-N/ZIS-Z has excellent stability.

## 4. Discussion

### Possible Mechanism of Photocatalytic Activity

**Figure 10:**
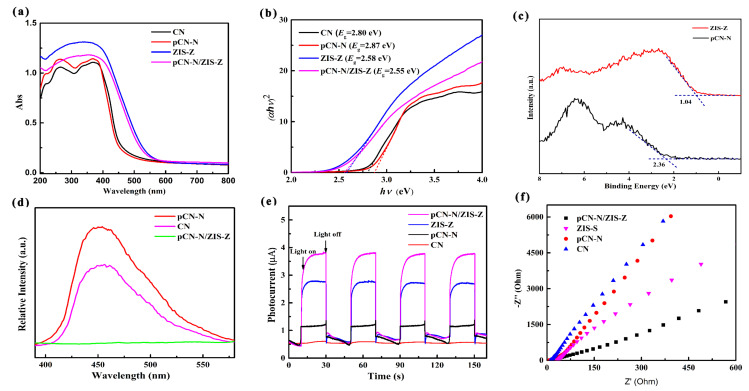
The UV–vis diffuse reflectance spectra (**a**), Plot of (*αhν*)^2^ vs. *hν* for the bandgap energy (**b**) of different photocatalysts. XPS valence band spectra of ZIS-Z and pCN-N (**c**). The photoluminescence spectra (**d**), transient photocurrent responses (**e**), and electrochemical impedance spectroscopy (**f**) of different photocatalysts.

Ultraviolet visible diffuse reflectance spectroscopy was used to explore the energy band structure and light absorption characteristics (Figure 10a). The absorption edges of pCN-N and CN were similar. It was not difficult to find that ZIS-Z shows a favorable visible-light absorption ability, because the zinc vacancies change the bandgap and expand the visible light absorption range. After pCN-N and ZIS-Z are combined, the light absorption range is between 200 nm and 800 nm and distinctly red-shifted compared with CN or pCN-N. The optical absorption bandgaps of CN, pCN-N, ZIS-Z and pCN-N/ZIS-Z nanocomposite are depicted in the Figure 10b. The values of energy bandgaps are calculated via the Kubelka–Munk method (the Kubelka–Munk formula: *αhν* = A (*hν* − *E*_g_) *^n^*^/2^) [44,62]. The bandgap values of CN, pCN-N, ZIS-Z and pCN-N/ZIS-Z are approximately 2.80 eV, 2.87 eV, 2.58 eV and 2.55 eV, respectively. By measuring the XPS valence band spectrum, the valence band energies (*E*_VB_) of pCN-N and ZIS-Z are 2.36 eV and 1.04 eV (Figure 10c), respectively. Therefore, it can be calculated that the conduction band energies (*E*_CB_) of pCN-N is –0.51eV and ZIS-Z is –1.54 eV by the equation *E*_CB_ = *E*_VB_ − *E*_g_.

Photoluminescence (PL) spectroscopy was used to explore the separation and transfer ability of the photogenerated electron-hole pairs of pCN/ZIS. As displayed in Figure 10d, PL spectroscopy was performed for CN, pCN-N, and pCN-N/ZIS-Z at room temperature_._ The fluorescence intensity of the pCN-N is weaker than CN, which is possibly due to the introduction of nitrogen defections and oxygen-doping [76,77]. Nitrogen vacancies and oxygen-doping improved the performance to catch electrons, the service life of charges was extended, and then the use of photogenerated charges was promoted in the redox procedure. The fluorescence intensity of the pCN-N/ZIS-Z nanocomposite is weakest; on the one hand, the zinc vacancies further extend the service life of charges by increasing the ability to capture electrons; on the other hand, the restructuring of photogenerated carriers is inhibited by forming a pCN-N/ZIS-Z heterojunction. Thus, the PL results showed that nitrogen vacancies, oxygen-doping and the formation of a heterojunction are beneficial to promote photocatalytic degradation under visible light.

Photoelectrochemical measurements show the relative separation and transfer ability of photogenerated carriers. The photocurrent density of pCN-N/ZIS-Z is much higher than CN, pCN-N and ZIS-Z with a hierarchical order of pCN-N/ZIS-Z > ZIS-Z > pCN-N > CN, revealing the effective separation and transportation of photogenerated carriers for pCN-N/ZIS-Z. Figure 10e displays the photocurrent densities of CN, pCN-N, ZIS-Z, and pCN-N/ZIS-Z with a sequence of pCN-N/ZIS-Z > ZIS-Z > pCN-N > CN. The changes of the current density are due to the separation efficiency of photogenerated charges. The electrochemical impedance spectroscopy (EIS) Nyquist plots are depicted in Figure 10f shows pCN-N/ZIS-Z has the smallest arc with a hierarchical order of pCN-N/ZIS-Z < ZIS-Z < pCN-N < CN. Based on the above analysis of PL and photoelectrochemical measurements, there is no doubt that the photoelectric performance can be significantly elevated through introducing defections, oxygen-doping, and the construction of the heterojunction in pCN-N/ZIS-Z.

**Figure 11:**
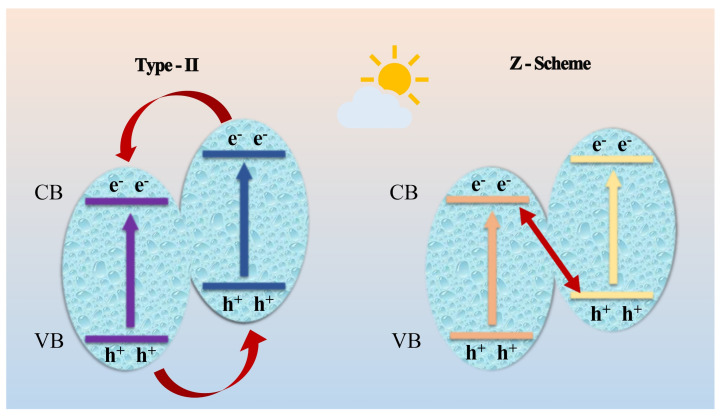
The electrons and holes transfer paths of two heterojunctions.

Figure 11 shows common heterojunction structures including type-II and Z-scheme heterojunctions [78]. As shown in Figure 11, we first make a conjecture that if pCN-N and ZIS-Z form a type-II heterojunction, photogenerated holes on the VB of pCN-N will move to the VB of ZIS-Z and electrons on the CB of pCN-N will react with dissolved O_2_ to form **·**O_2_^−^. However, the potential of the holes remaining on the VB of ZIS-Z is +1.04 eV, which is lower than the normal potential of the H_2_O/**·**OH couple (+2.27 eV). Then, the holes remaining on the VB of ZIS-Z cannot be oxidized to generate **·**OH. In the capture experiment, **·**OH is an active free radical, the conclusion being contrary to the capture experiment result. It can be found that the holes (+2.36 eV) left on the VB of pCN-N can be oxidized to generate **·**OH. At the same time, the holes left on the VB of pCN-N can also directly participate in a photocatalytic reaction. It can be further inferred that the holes on the VB of pCN-N will not be transferred to the VB of ZIS-Z. The electrons on the CB of ZIS-Z and the holes remaining on the VB of pCN-N participate in the catalytic degradation of pollutants through redox reactions. Based on the capture experiments and conjectures, a conclusion can be drawn that pCN-N/ZIS-Z is a Z-scheme heterojunction.

**Figure 12:**
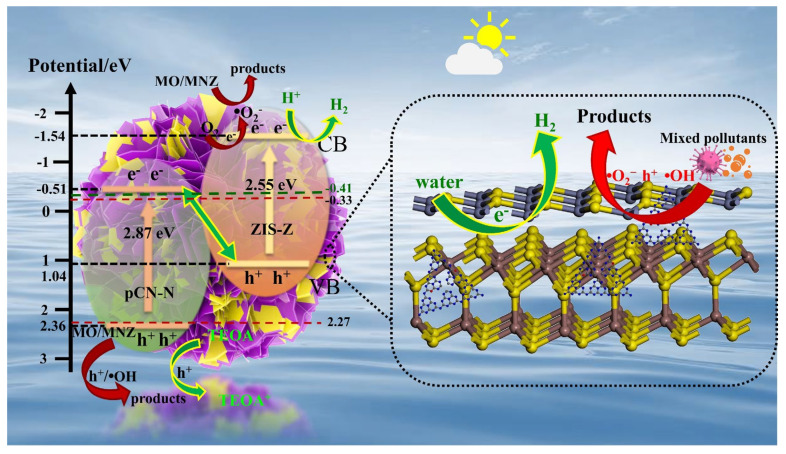
The possible photocatalytic mechanism of pCN-N/ZIS-Z.

Figure 12 exhibits the possible mechanism of photocatalytic hydrogen evolution and degradation of mixed pollutants. When a certain energy of light (λ ≥ 420 nm) is illuminated on pCN-N/ZIS-Z, the electrons on the valence band are excited and transferred to the conduction band, thus leaving the corresponding holes on the valence band, Equation (1). In the hydrogen production experimental system, since pCN-N/ZIS-Z has a Z-scheme heterojunction and the conduction band (CB) potential of ZIS-Z is more negative, electrons in the CB of pCN-N will migrate to ZIS-Z quickly. As a result, the photogenerated electron-hole pairs in the heterojunction are efficiently separated. In addition, compared with a single component, the recombination of photogenerated electron-hole pairs is obviously inhibited. The electrons on the CB of ZIS-Z are reductive and directly react with H^+^ in the water to produce H_2_ (−0.41 V vs. NHE). As a co-catalyst, Pt carries a large number of electrons to react with H^+^ to produce H_2_ (2H_2_O + 2e^−^ → H_2_ + 2OH^−^). On the other hand, in order to prevent oxidation, TEOA acts as a sacrificial agent to capture h^+^ in the pCN-N valence band (VB) and quench them in time (TEOA + h^+^ → TEOA^+^). The efficient separation and transfer of electron-hole pairs significantly improve the hydrogen evolution performance of the catalyst. This possible catalytic mechanism is consistent with the results of photocatalysis experiments and the wavelength-dependent AQY spectrum. In the process of carrier migration, the internal electric field formed by the interface between pCN-N and ZIS-Z provides a continuous driving force for the photocatalytic hydrogen evolution reaction. In addition, because the morphology of pCN-N/ZIS-Z is a nano-sheet porous structure, compared with a single component, the specific surface area of the catalyst is enlarged and more active sites are exposed, which further improves the pCN-N/ZIS-Z photocatalytic performance of hydrogen evolution.

In the photocatalytic degradation experiment, the CB edges of ZIS-Z (−1.54 eV) are higher than the normal potential of the **·**O_2_^−^/O_2_ couple (−0.33 eV). Therefore, electrons on the CB of ZIS-Z could react with dissolved O_2_ to form **·**O_2_^−^ and **·**O_2_^−^ further reacts with pollutants, Equations (2) and (4). Analogously, the VB edges of pCN-N (+2.36 eV) are lower than the normal potential of the **·**OH/H_2_O couple (+2.27 eV). Then, the photogenerated holes tend to keep on the VB of pCN-N, and could also produce **·**OH by oxidizing H_2_O and **·**OH to further react with pollutants, Equations (3) and (4). Moreover, holes on the pCN-N valence band could directly oxidize pollutants, Equation (4). While the electrons transfer to the VB of ZIS-Z from the CB of pCN-N. The free radicals of **·**O_2_^−^, h^+^ and **·**OH can degrade MO and MNZ, Equation (4), which is consistent with the conclusion of the free radical capture experiment. Therefore, it can be determined that the Z-scheme heterojunction is established between pCN-N and ZIS-Z. Based on the above analysis, the probable processes for MO/MNZ degradation can be summarized in Equations (1)–(4):

(1) $$\mathrm{pCN}-N/\mathrm{ZIS}-Z+h\nu \to h^{+}+e^{-}$$

(2) $$e^{-}+O_{2}\to \cdot O_{2}^{-}$$

(3) $$h^{+}+{\mathrm{OH}}^{-}/H_{2}O\to \cdot \mathrm{OH}$$

(4) $$O_{2}^{-}/h^{+}/\cdot \mathrm{OH}+\mathrm{MO}/\mathrm{MNZ}\to \mathrm{Products}$$

## 5. Conclusions

Oxygen-doped pCN-N/ZIS-Z based on defect engineering is synthesized for highly efficient degradation of mixed pollutants and hydrogen production under visible light. The hydrogen production of pCN-N/ZIS-Z (9189.8 µmol·h^−1^·g^−1^) is 58.9 times that of CN (155.9 µmol·h^−1^·g^−1^) and 1.2 times that of ZIS-Z (7575.4 µmol·h^−1^·g^−1^). The pCN-N/ZIS-Z achieved a catalytic efficiency of 98.7% and 92.5% for MO and MNZ in the mixed pollutants, respectively. The reason why pCN-N/ZIS-Z has excellent photocatalytic degradation and hydrogen production performance is mainly due to the following: (1) nanosheets with porous structures expand the surface area of the catalyst and provide more active sites for photocatalytic reactions. (2) The construction of the Z-scheme heterojunction improved the separation efficiency of photogenerated charges. (3) Introduction of defect engineering and oxygen-doping tend to trap electrons on the surface of pCN-N/ZIS-Z to reduce interior charge recombination, thereby generating more active participators for photocatalytic reactions. The degradation efficiency after four cycles almost invariability demonstrated the excellent stability of the pCN-N/ZIS-Z nanocomposite. Based on the above characteristics, the pCN-N/ZIS-Z photocatalyst was prospectively applied in practice, and this research provides ideas for studying multifunctional catalysts to solve the energy crisis and for environmental restoration.

## Figures and Tables

**Table 1:** The comparison of our work with the results of other similar investigations.

Catalyst	Light Source	H_2_ (µmol·h^−1^·g^−1^)	Ref.
NH_2_-MIL-125/g-C_3_N_4_	300W Xe (λ > 400 nm)	1123	[68]
MoS_2_/g-C_3_N_4_	300W Xe (λ > 400 nm)	1155	[69]
g-C_3_N_4_@ZnIn_2_S_4_	300W Xe (λ > 400 nm)	2780	[70]
ZnIn_2_S_4_/g-C_3_N_4_	300W Xe (λ > 400 nm)	3870	[71]
BCN/AZIS	300W Xe (λ > 400 nm)	4854	[44]
pCN-N/ZIS-Z	300W Xe (λ > 420 nm)	9189.8	This work

## Data Availability

Not applicable.

## Supplementary Materials

The following are available online at https://www.mdpi.com/article/10.3390/nano11102483/s1. Table S1: S_BET_ and Pore Parameters of Catalyst, Figure S1: The molecular structures of MNZ (a) and MO (b), Figure S2: The N_2_ adsorption-desorption isotherms of CN and pCN-N, Figure S3: EPR spectra of CN, pCN-N (a) ZIS and ZIS-Z (b) samples, Figure S4: The parallel experiments of hydrogen production, Figure S5: HPLC chromatograms of MO (a) and MNZ (b) change with reaction time, Figure S6: Raman spectra of MNZ (30 mg/L) before and after degradation.
