# Supplementary material for: Flower-Like Dual-Defective Z-Scheme Heterojunction g-C3N4/ZnIn2S4 High-Efficiency Photocatalytic Hydrogen Evolution and Degradation of Mixed Pollutants
Source: Nanomaterials (Basel). 2021 Sep 24;11(10):2483. doi: 10.3390/nano11102483 (PMC8539809; doi:10.3390/nano11102483)
Supplement: Supplementary file 1 [file nanomaterials-11-02483-s001.zip › nanomaterials-1376284-supplementary.pdf]

nanomaterials

Article

Supporting Information

Flower-like dual-defective Z-scheme heterojunction g-C_3N_4/$ZnIn_2S_4$ high-efficiency photocatalytic hydrogen evolution and degradation of mixed pollutants

Linlin Hou [1,2,†], Zhiliang Wu [1,2,†], Chun Jin [1,2], Wei Li [1,2,*], Qiuming Wei [1,2], Yasi Chen [1,2] and Teng Wang [3]

1 Guangdong Provincial Key Laboratory of Quantum Engineering and Quantum Materials, Guangdong Engineering Technology Research Center of Efficient Green Energy and Environmental Protection Materials, School of Physics and Telecommunication Engineering, South China Normal University, Guangzhou 510006, China; 2019021952@m.scnu.edu.cn (L.H.); 2019021957@m.scnu.edu.cn (Z.W.); chunjin@m.scnu.edu.cn (C.J.); 2019021930@m.scnu.edu.cn (Q.W.); yasi@m.scnu.edu.cn (Y.C.)
2 Guangdong Provincial Key Laboratory of Nuclear Science, South China Normal University, Guangzhou 510006, China
3 School of Computer Science, South China Normal University, Guangzhou 510006, China; towangteng@263.net
* Correspondence: liwei@m.scnu.edu.cn; Tel.: +86-020-39310066
† These authors contributed equally to this work and should be considered co-first authors.

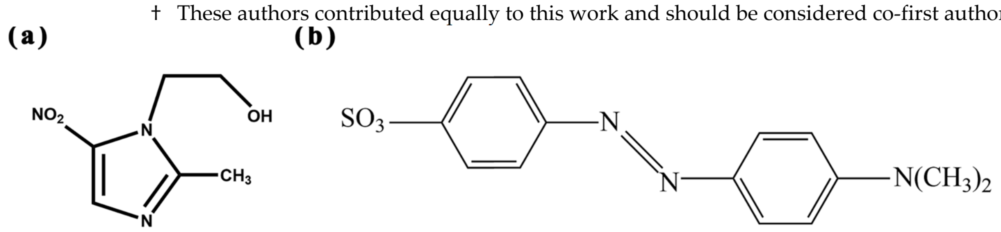

Figure S1. The molecular structures of MNZ (a) and MO (b).

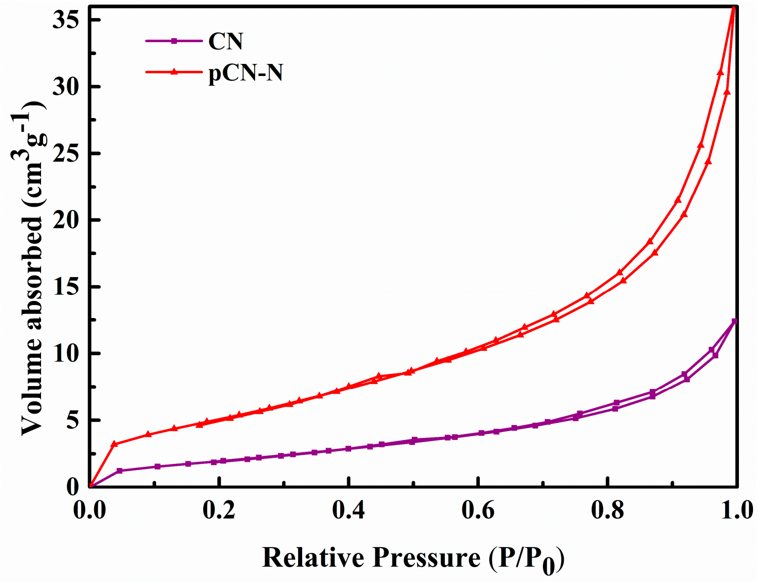

Figure S2. The N_2 adsorption-desorption isotherms of CN and pCN-N.

Table S1. SBET and Pore Parameters of Catalyst.

Catalyst	S_{BET} (m^2/g)	average pore diameter (nm)	pore volume (cm^3/g)
CN	7.837	9.783	0.019
pCN-N	19.843	11.360	0.056
ZIS-Z	74.691	10.805	0.202
pCN-N/ZIS-Z	81.848	12.833	0.263

The electron paramagnetic resonance (EPR) measurement was executed to evaluate the subsistence of the vacancies. Figure. S1a shows the strong symmetrical resonance signal at g = 2.0035. For CN, the Lorentzian line is generated due to the single electron in the carbon atom [1, 2]. The intensity of the Lorentzian line for the pCN-N is immensely enhanced because of the increase of unpaired electrons engendered from the introduction of nitrogen vacancies. Generally speaking, When the nitrogen vacancies were generated in CN, extra electrons will be quickly reallocated to their nearest carbon atoms by the delocalized π-conjugated networks of CN [3-5]. Analogously, ZIS and ZIS-Z exhibit strong symmetrical resonance signal at g = 2.0032 (Figure. S1b). ZIS-Z has a higher intensity than ZIS, which illustrates ZIS-Z possess zinc vacancies [6]. According to the above data, it can be considered that there are nitrogen vacancies in pCN-N and zinc vacancies in ZIS-Z. Vacancies can restrain the recombination of electron-hole pairs, which is conducive to photocatalytic degradation.

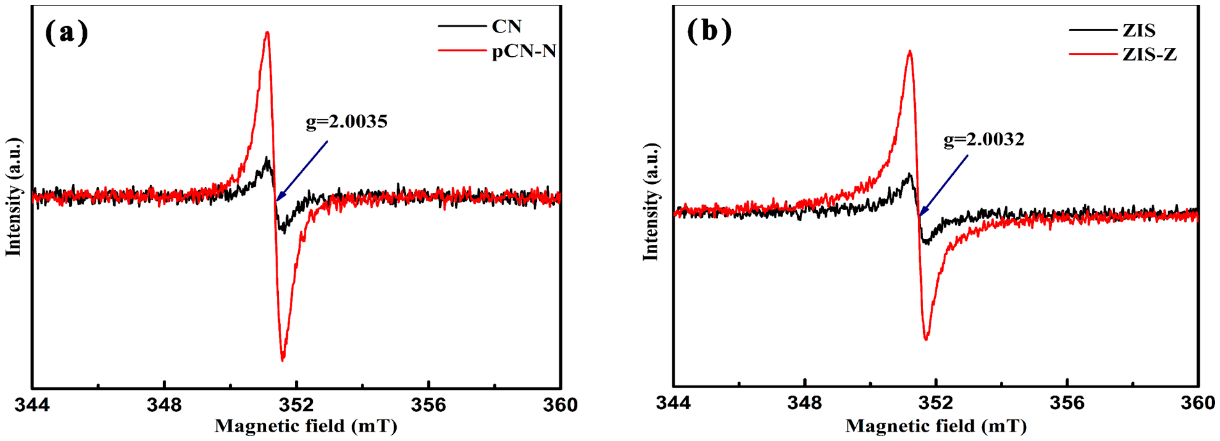

Figure S3. EPR spectra of CN, pCN-N (a) ZIS and ZIS-Z (b) samples.

The apparent quantum yield (AQY) was measured by inserting an appropriate band-pass filter on the light source. Other conditions are similar to those for hydrogen measurement. The AQY of 365 nm, 420 nm, 450 nm, and 500 nm wavelengths are 10.57%, 8.71%, 3.66%, and 1.59% respectively. The calculation is as follows:

AQY = [(Number of evolved hydrogen molecules × 2) / Number of incident photons] × 100%

$$AQY = \frac{N_e}{N_p} \times 100\% = \frac{2 \times M \times N_A}{\frac{E_{total}}{E_{photon}}} \times 100\% = \frac{2M \times N_A}{\frac{S \times P \times t}{h \times \frac{c}{\lambda}}} \times 100\% = \frac{2 \times M \times N_A \times h \times c}{S \times P \times t \times \lambda} \times 100\%$$

Where M is the production of hydrogen (mol), N_A is Avogadro constant (6.022×10^{23}/mol), h is Planck constant (6.626×10^{-34} J·s), c is the speed of light (3×10^8 m/s), S is

the irradiation area (cm²), P is the intensity of irradiation light (W/cm²), t is the photoreaction time (s), λ is the wavelength of the monochromatic light (m) [7].

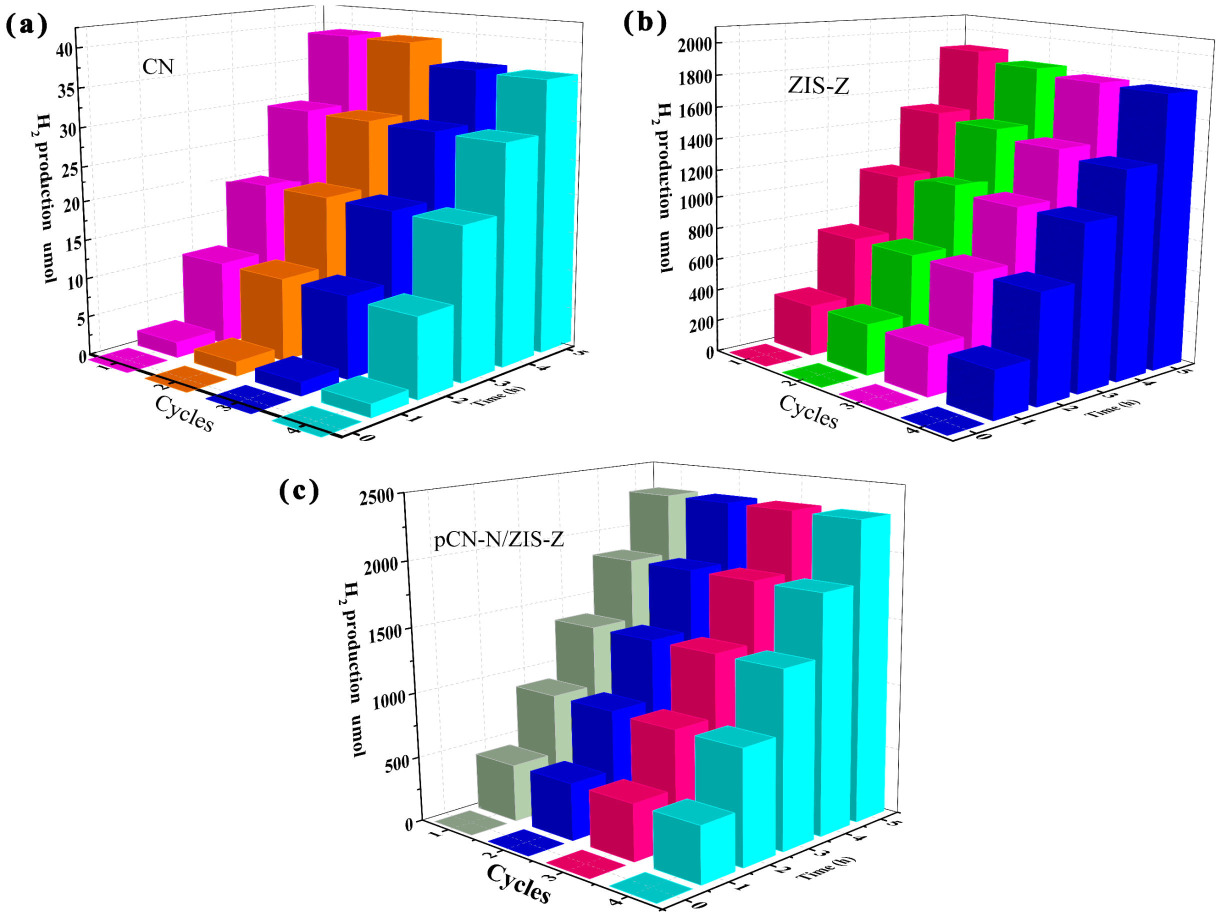

Figure S4. The parallel experiments of hydrogen production.

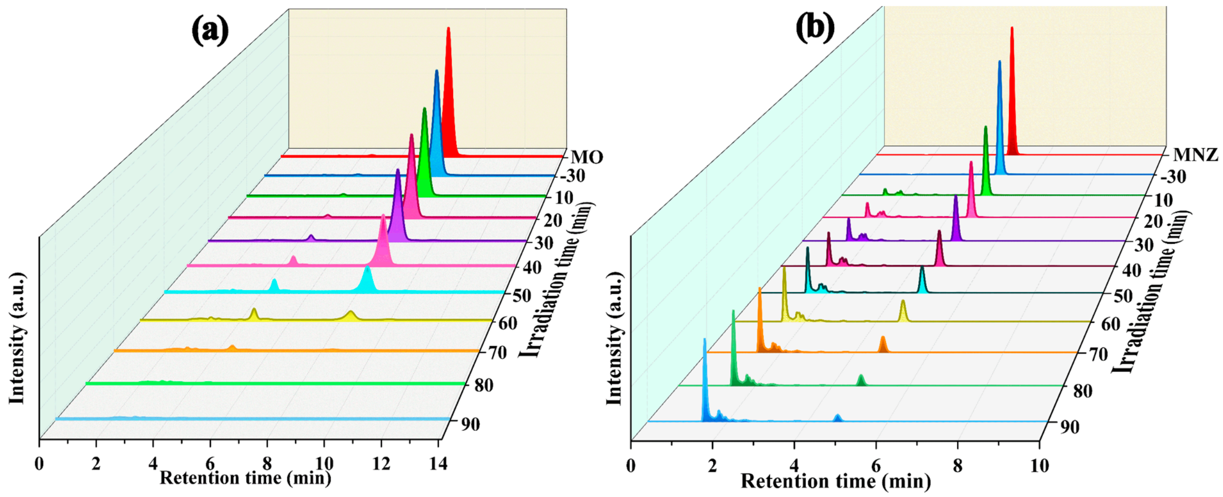

Figure S5. HPLC chromatograms of MO (a) and MNZ (b) change with reaction time.

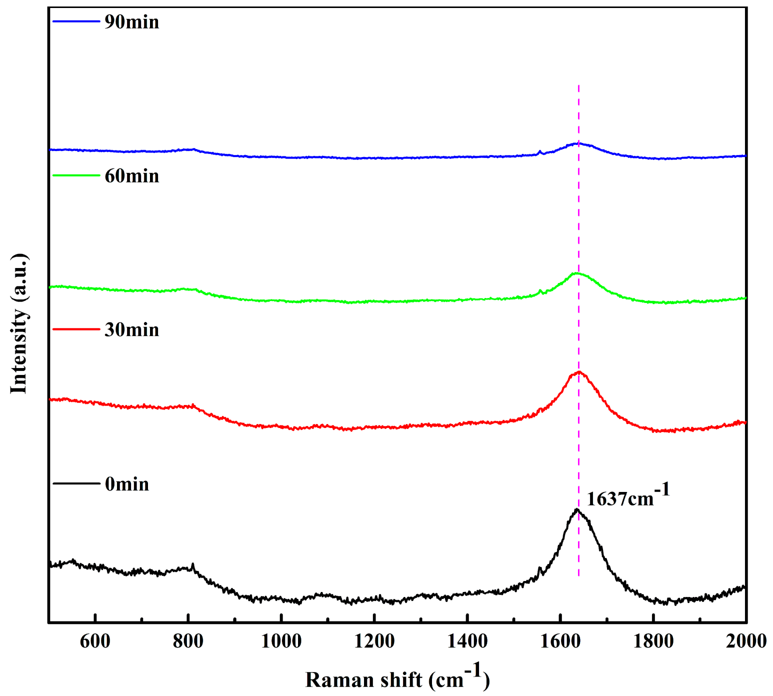

Figure S6. Raman spectra of MNZ (30mg/L) before and after degradation.

References

1. X. Jiao, Z. Chen, X. Li, Y. Sun, S. Gao, W. Yan, C. Wang, Q. Zhang, Y. Lin, Y. Luo, Y. Xie, Defect-Mediated Electron-Hole Separation in One-Unit-Cell $ZnIn_2S_4$ Layers for Boosted Solar-Driven CO_2 Reduction, J Am Chem Soc, 139 (2017) 7586-7594.
2. L. Kong, X. Mu, X. Fan, R. Li, Y. Zhang, P. Song, F. Ma, M. Sun, Site-selected N vacancy of g-C_3N_4 for photocatalysis and physical mechanism, Applied Materials Today, 13 (2018) 329-338.
3. G. Wu, L. Yu, Y. Liu, J. Zhao, Z. Han, G. Geng, One step synthesis of N vacancy-doped g-C_3N_4/Ag_2CO_3 heterojunction catalyst with outstanding "two-path" photocatalytic N2 fixation ability via in-situ self-sacrificial method, Applied Surface Science, 481 (2019) 649-660.
4. P. Yang, H. Zhuzhang, R. Wang, W. Lin, X. Wang, Carbon Vacancies in a Melon Polymeric Matrix Promote Photocatalytic Carbon Dioxide Conversion, Angew Chem Int Ed Engl, 58 (2019) 1134-1137.
5. Y. Xue, Y. Guo, Z. Liang, H. Cui, J. Tian, Porous g-C_3N_4 with nitrogen defects and cyano groups for excellent photocatalytic nitrogen fixation without co-catalysts, Journal of Colloid and Interface Science, 556 (2019) 206-213.
6. Y. Qin, H. Li, J. Lu, Y. Feng, F. Meng, C. Ma, Y. Yan, M. Meng, Synergy between van der waals heterojunction and vacancy in $ZnIn_2S_4$/g-C_3N_4 2D/2D photocatalysts for enhanced photocatalytic hydrogen evolution, Applied Catalysis B: Environmental, 277 (2020).
7. A.M. Elewa, M.H. Elsayed, A.F.M. El-Mahdy, C.-L. Chang, L.-Y. Ting, W.-C. Lin, C.-Y. Lu, H.-H. Chou, Triptycene-based discontinuously-conjugated covalent organic polymer photocatalysts for visible-light-driven hydrogen evolution from water, Applied Catalysis B: Environmental, 285 (2021) 119802.
